# Diabetes‐mediated promotion of colon mucosa carcinogenesis is associated with mitochondrial dysfunction

**DOI:** 10.1002/1878-0261.12531

**Published:** 2019-07-27

**Authors:** Laura Del Puerto‐Nevado, Aranzazu Santiago‐Hernandez, Sonia Solanes‐Casado, Nieves Gonzalez, Marta Ricote, Marta Corton, Isabel Prieto, Sebastian Mas, Ana Belen Sanz, Oscar Aguilera, Carmen Gomez‐Guerrero, Carmen Ayuso, Alberto Ortiz, Federico Rojo, Jesus Egido, Jesus Garcia‐Foncillas, Pablo Minguez, Gloria Alvarez‐Llamas, on behalf of the DiabetesCancerConnect Consortium

**Affiliations:** ^1^ Translational Oncology Division Oncohealth Institute, IIS‐Fundacion Jimenez Diaz‐UAM Madrid Spain; ^2^ Immunology Department IIS‐Fundacion Jimenez Diaz‐UAM Madrid Spain; ^3^ Renal, Vascular and Diabetes Research Laboratory, Spanish Biomedical Research Network in Diabetes and Associated Metabolic Disorders (CIBERDEM) IIS‐Fundacion Jimenez Diaz‐UAM Madrid Spain; ^4^ Genetics Department IIS‐Fundacion Jimenez Diaz‐UAM Madrid Spain; ^5^ Center for Biomedical Network Research on Rare Diseases (CIBERER) ISCIII Madrid Spain; ^6^ Radiation Oncology Oncohealth Institute, IIS‐Fundacion Jimenez Diaz‐UAM Madrid Spain; ^7^ Nephrology and Hypertension Department IIS‐Fundacion Jimenez Diaz‐UAM Madrid Spain; ^8^ REDINREN Madrid Spain; ^9^ Pathology Department IIS‐Fundacion Jimenez Diaz‐UAM Madrid Spain

**Keywords:** colon cancer, diabetes, field of cancerization, mitochondria, proteomics

## Abstract

Type 2 diabetes mellitus (T2DM) has been associated with an increased risk of cancer, including colon cancer (CC). However, we recently reported no influence of T2DM on CC prognosis, suggesting that any effect might be at the early stages of tumor development. We hypothesized that T2DM may create an environment in the healthy tissue, which acts as a carcinogenesis driver in agreement with the field of cancerization concept. Here, we focused on early carcinogenesis by analyzing paired tumor and normal colonic mucosa samples from the same patients. The proteome of CC and paired mucosa was quantitatively analyzed in 28 individuals (12 diabetics and 16 nondiabetics) by mass spectrometry with isobaric labeling. Out of 3076 identified proteins, 425 were differentially expressed at the tumor in diabetics compared with nondiabetics. In the adjacent mucosa, 143 proteins were differentially expressed in diabetics and nondiabetics. An enrichment analysis of this signature pointed to mitochondria, ribosome, and translation. Only six proteins were upregulated by diabetes both in tumor and mucosa, of which five were mitochondrial proteins. Differential expression in diabetic versus nondiabetic mucosa was confirmed for MRPL53, MRPL18, and TIMM8B. Higher levels of MRPL18, TIMM8B, and EIF1A were also found in normal colon epithelial cells exposed to high‐glucose conditions. We conclude that T2DM is associated with specific molecular changes in the normal mucosa of CC patients, consistent with field of cancerization in a diabetic environment. The mitochondrial protein signature identifies a potential therapeutic target that could underlie the higher risk of CC in diabetics.

**Abbreviations**
ADA: American diabetic association
CC: colon cancer
FC: field cancerization
FDR: false discovery rate
LC‐MS/MS: liquid chromatography with mass spectrometry in tandem
N: normal mucosa (nondiabetic)
ND: normal mucosa diabetic
PSMs: peptide spectra matched
T: tumor (nondiabetic)
T2DM: type 2 diabetes mellitus
TD: tumor diabetic

## 1 Introduction

Type 2 diabetes mellitus (T2DM) is a known risk factor for a wide spectrum of pathological disorders such as hypertension and cardiovascular diseases. Epidemiologic evidence suggests an association between T2DM and increased risk of many forms of cancer (Johnson *et al.*, 2012; Renehan *et al.*, 2012; Suh and Kim, 2011) including colon cancer (CC) (Harding *et al.*, 2015; de Kort *et al.*, 2016). In a recent publication, we reported no differences in CC prognosis once the tumor is present (Prieto *et al*., 2017). Thus, the challenge is to focus on the role of T2DM during early carcinogenesis, an stage which has obvious limitations in humans from the point of view of research approaches. In the omics era, an unexplored hypothesis like this requires the production of extensive repositories of molecular data that allows an unbiased exploration of molecular markers.

The existence of a differential proteome between tumor tissue and noncancer tissue has been widely reported (Cardoso *et al.*, 2007; Jimenez *et al.*, 2010; Nambiar *et al.*, 2010). Changes in the proteome of the normal mucosa adjacent to the tumor have also been shown, in agreement with the ‘field cancerization’ (FC) concept (Curtius *et al.*, 2018; Slaughter *et al.*, 1953). The concept of FC conforms a theoretical framework for carcinogenesis. This theory defines premalignant epithelial areas with normal histology, but with specific molecular features that could promote cancer development. Specifically, in colorectal cancer, several authors have reported evidences regarding a ‘field effect’ in the normal mucosa surrounding the tumor characterized by changes in methylation pattern, chromosomal instability, copy number alterations, or even in Warburg metabolism. Among the potential mechanisms responsible for this effect, T2DM has been proposed as a factor for FC in *in vitro* approaches (Del Puerto‐Nevado *et al.*, 2019; Rubin, 2013; Slaughter *et al.*, 1953). Gene and protein deregulation has been reported in such ‘normal’ tissue (Guo *et al.*, 2017; Sanz‐Pamplona *et al.*, 2014), and gradual signatures were correlated with distance to the tumor. At the protein level, 1808 proteins showed significant variation between normal and colorectal cancer tissue, constituting a much larger fraction than that reported at the transcriptome level (Wiśniewski *et al.*, 2012). An extensive proteomics analysis comparing normal tissue, adenoma, and CC tissue found more than 2000 proteins altered, and main changes related to fatty acid metabolism and plasma membrane transport (Wiśniewski *et al.*, 2015). Supporting the FC hypothesis, shotgun proteomics previously disclosed that colorectal precancerous lesions (adenomas) and a paired sample of normal colon mucosa displayed protein differences that had been reported only in advanced cancer stages (Uzozie *et al.*, 2014).

In agreement with these evidences, we recently showed how T2DM could promote a precancer state by means of signaling pathways exclusively activated in diabetic normal mucosa (Del Puerto‐Nevado *et al.*, 2019). Molecular changes specifically occurring in the normal colon mucosa of diabetic patients would thus explain their higher risk of CC development. In this study, we present a quantitative differential proteomics analysis of tumor tissue and adjacent normal mucosa from CC cancer patients with and without T2DM. We focused on molecular changes in patient‐matched normal mucosa from diabetics and nondiabetics to identify potential proteins changes promoted by diabetes that may drive CC development. Alterations in this ‘healthy’ area would mimic an earlier stage in cancer progression compared with the tumor, revealing novel pathogenetic mechanisms and potential targets for drug design.

## 2 Methods

### 2.1 Patient selection

Patients were recruited from January 2009 to December 2013 at the University Hospital Fundación Jiménez Díaz (Madrid, Spain), a cohort used in a previous epidemiological study (Prieto *et al*., 2017). The inclusion parameters were as follows: patients with resection of primary CC, colon adenocarcinoma histological type, colon location (rectal cancer patients were excluded as they were receiving chemo/radiotherapy), time from surgery up to 6 months, no neoadjuvant treatment, no other concurrent neoplasia or immunosuppressive treatment, and diabetes diagnosed as a documented registry of diabetes, history of antidiabetic medication, or meeting the American Diabetic Association (ADA) criteria for diabetes at the time of reviewing the data. The ADA criteria for diabetes diagnosis were hemoglobin A1c values ≥ 6.5%, or fasting blood glucose levels ≥ 125 mg·dL^−1^ with high fasting values recorded at least twice, or random blood glucose levels ≥ 200 mg·dL^−1^ with high random values recorded at least twice. In parallel, this observational study also included 79 nondiabetic patients with primary diagnosis of CC, who underwent resection during the same period, using equal inclusion criteria except the presence of diabetes, aiming to obtain a well‐balanced series. A total of 28 patients (12 diabetics and 16 nondiabetics) that met the inclusion criteria described above were selected based on sample availability and homogeneity criteria to compare a well‐balanced subset of patients, in terms of: gender, grade [low grade: G1–G2 and high grade: G3, following the 2010 WHO classification (http://www.pathologyoutlines.com/topic/colontumorwhoclassification.html)], tumor site (right: cecum, hepatic flexure, ascending, and transverse colon; and left: splenic flexure and descending colon), stage (low stage: 0, I, any II; or high stage: any III, IV; https://cancerstaging.org/Pages/default.aspx), recurrence, death, or alive status at final follow‐up and metformin intake. The study was approved by the Institutional Scientific and Ethical Committee at IIS‐Fundación Jiménez Díaz (Madrid, Spain; CEIC‐FJD, approval code 08/13; on October 1, 2013) in accordance with the ethical principles stated in the Declaration of Helsinki. Informed consent is included in the clinical history of each participant and recorded by the standard requirements of data protection rules.

### 2.2 Tissue sampling

Surgical resection specimens from CC tumors were obtained from Fundacion Jimenez Diaz Biobank. Paired formalin‐fixed paraffin‐embedded (FFPE) samples from tumor and nontumor adjacent normal colonic mucosa from each individual were selected. Cancer tissue was obtained from the resected tumor edge, and the percentage of tumor content in FFPE samples was more than the 70%. Normal colonic mucosa samples were selected from a > 5‐cm distance from the tumor. Pathologists confirmed the absence of morphological lesions in the normal colonic tissue.

### 2.3 Proliferation, microsatellite instability phenotype, and *RAS* and *BRAF* mutational analysis

Mutational analysis for *BRAF, KRAS, and NRAS* genes was performed on FFPE CC samples by pyrosequencing and PCR‐based assay. DNA was isolated from 20‐µm FFPE sections of representative tumor tissue. *KRAS* and *NRAS* were studied by pyrosequencing using the therascreen *KRAS* and *RAS* Extension Pyro Kits (Qiagen, Venlo, the Netherlands), following the manufacturer’s recommendations. *BRAF* was assayed by the PCR‐based Cobas 4800 BRAF V600 Mutation Test (Roche, Basel, Switzerland). The microsatellite instability (MSI) phenotype was studied by testing the expression of the four mismatch repair (MMR) proteins (MLH1, MSH2, MSH6, PMS2) by immunohistochemistry on an Omnis platform (Dako, Glostrup, Denmark), using conventional 3‐µm tissue sections from the same specimens. Interpretation of staining was performed by qualified pathologists. Finally, proliferation was estimated as percentage Ki67‐labeled tumor cells by immunohistochemistry on an Omnis platform.

### 2.4 Differential protein analysis by isobaric labeling and LC‐MS/MS

Ten slices 5 µm thick were collected from each FFPE sample. Tissue was deparaffinated and proteins extracted as previously described (Gámez‐Pozo *et al.*, 2012). Total protein was quantified by the BCA Protein Assay Kit (Thermo Scientific, Waltham, MA, USA). Two biological replicates were analyzed per condition, diabetic and nondiabetic tumor tissue (TD and T, respectively), and their adjacent diabetic and nondiabetic normal mucosa (ND and N, respectively; Fig. S1). Each biological replicate was a pool composed by eight individual samples (in the case of nondiabetics, T or N) or six individual samples (in the case of diabetics, TD or ND). Pools were designed to minimize any bias which could interfere with the identification of differences uniquely attributed to diabetes; that is, every pool contained 50% of samples from patients taking insulin and 50% of patients taking metformin and every pool contained samples from patients with both right and left side tumors. Digestion was performed using the filter‐aided sample preparation method (Wiśniewski *et al.*, 2009). A total of 75 µg of each tryptic digest was labeled according to the manufacturer's instructions (AB Sciex, Redwood City, CA, USA) with one 8‐plex isobaric amine‐reactive tag per cell line iTRAQ® Reagent 8‐plex kit (AB Sciex, Redwood City, CA, USA). Labeled samples were combined, cleaned up using a Sep‐Pak C18 cartridge for SPE (Waters Corp., Milford, MA, USA), and fractionated using high pH reverse phase technique (Wang *et al.*, 2011). All samples were analyzed by liquid chromatography with mass spectrometry in tandem (LC‐MS/MS) on The LTQ Orbitrap Velos mass spectrometer (Thermo Scientific) coupled to an Eksigent nano LC system (Eksigent, Redwood City, CA, USA) through a nanoelectrospray ion source (Proxeon Biosystems, Odense, Denmark). Separation took place in a binary gradient of 4% ACN in 0.1% FA (buffer A) and 100% ACN in 0.1% FA (buffer B), with a flow rate of 250 nL·min^−1^, as follows: 0 − 2 min 6% B, 2 − 133 min 30% B, and 133 − 143 min 98% B. The LTQ Orbitrap Velos was operated in positive ionization mode. The resolution was set to 30 000 FWHM at *m*/*z* 400. HCD was used for fragmentation; up to the 15 most abundant isotope patterns with charge ≥ 2 from the survey scan were selected for fragmentation in the HCD collision cell. Data files were analyzed using proteome discoverer 1.4 (Thermo Scientific) with Sequest HT as the search engine against a concatenated Uniprot database of Homo sapiens (20 187 sequences) supplemented with frequently observed contaminants (397 sequences). Reagent impurities were corrected as indicated by the manufacturer. Peptide spectral matches (PSMs) were filtered using Percolator with a false discovery rate (FDR) of 1%. Quantification results at the PSM level were exported for further analysis. Quantification and statistical analysis were performed using Isobar in R. We used a noise model that accounts for the technical variation due to the instrument. A null protein distribution was used to model sample variability (created by comparing biological replicates). Protein ratios were further calculated for all the possible combinations, and only, proteins having both ‘*P*‐value sample’ and ‘*P*‐value ratio’ under 5% were considered significant.

### 2.5 Enzyme‐linked immunosorbent assays

For ELISA analysis of proteins MRPL18 (SEP261Hu; Cloud Clone Corp, Houston, TX, USA), MRPL53 (SEP070Hu; Cloud Clone Corp), and TIMM8B (CSB‐EL023558HU; Cusabio, Houston, TX, USA) manufactures’ protocols were followed.

### 2.6 Cell culture

The epithelial cell line NCM356 derived from normal colon mucosa (acquired under a MTA from InCell Corp., San Antonio, TX, USA) was selected to arrange an *in vitro* validation.

To mimic the diabetic and nondiabetic environment of normal mucosa used for proteomics, cells were exposed to three experimental conditions: normoglycemia (cells were cultured in serum‐free M3Base™ medium (Incell Corp.) supplemented with 10% FBS and 1% penicillin–streptomycin), high‐glucose condition (by adding 24.5 mm d‐glucose to M3Base™), and an osmotic control (by adding 24.5 nm l‐glucose to M3Base™) for five days. Then, cells were trypsinized at a 75–80% confluence and pellets were used for protein extraction.

### 2.7 Immunoblotting

Proteins were quantified using a BCA kit (Thermo Fisher), and 15 µg of protein from each condition was loaded into 10% polyacrylamide gels. After electrophoresis, proteins were transferred onto nitrocellulose membranes at 100 V for 2 h. Membranes were blocked and then incubated with primary antibodies against MRLP18 (1 : 100, SAB1400513; Sigma‐Aldrich, Darmstadt, Germany), TIMM8B (1 : 1000, H00026521‐M15; Novus Biological, Centennial, CO, USA), and EIF1A (1 : 1000, ab172623; abCAM, Cambridge, UK) overnight at 4 °C. After rinsing with Tris‐buffered saline with Tween (TBST), the corresponding secondary antibody was added and incubated for 1 h. An Amersham Imager 600 chemiluminescence imager was used for high‐resolution digital imaging of proteins, and the gray values of the target bands were analyzed with imagej software (NIH ImageJ, Washington D.C, USA). These experiments were carried out in triplicate. β‐actin (Sigma‐Aldrich) was used as loading control. Values obtained were normalized with β‐actin expression, and the expression of hyperglycemic and osmotic control proteins was referred to normoglycemic conditions. Differences among groups were studied by Mann–Whitney *U*‐test, and statistical significance was considered when *P* < 0.05.

### 2.8 Functional enrichment analyses

The functional enrichment analyses were performed using the Panther suite (Mi *et al.*, 2013). In all comparisons performed, the proteins with FDR < 0.05 and fold change > 1.2 were selected and submitted to the statistical overrepresentation test with all the proteins detected by the proteomics experiments as the reference set. In all analyses, we used the Fisher's exact test for the statistics and FDR for the multiple testing *P*‐value correction. Gene Ontology terms and Reactome pathways over‐represented with FDR < 0.05 were reported.

## 3 Results

### 3.1 Patient characteristics

From a Spanish cohort of 160 CC patients, we selected 28 patients (16 nondiabetics matched to 12 diabetics, Table 1). Paired FFPE samples of tumor and normal colonic mucosa were used for proteomic analysis without differences between diabetics and nondiabetics. To confirm an equal distribution among study groups and reduce potential bias which could limit data interpretation, we included the evaluation of pathological variables such *KRAS*, *NRAS,* and *BRAF* mutational status (these genes are commonly mutated in colorectal cancer, showing a strong association with therapy resistance and patient outcome), MMR genes expression: *MLH1, MSH2, MSH6,* and *PSM2* (their loss of expression is related to MSI), and tumor proliferation status based on Ki‐67 expression.

**Table 1:** Clinical characteristics of CC patients included in the study.

	Nondiabetic (*n* = 16)	Diabetic (*n* = 12)
Age (years)	69 ± 8	74 ± 6
Male gender	7	9
BMI (kg·m^−2^)	24 ± 4	26 ± 2
CC grade^a^		
Low grade	4	2
High grade	12	10
CC stage^b^		
G1–G2	13	7
G3	3	5
Tumor site		
Right	7	6
Left	9	6
Recurrence	2	3
Alive at last follow‐up	15	10
Metformin	0	6

a Low grade was defined as G1‐G2 and high grade as G3, following the 2010 WHO classification (http://www.pathologyoutlines.com/topic/colontumorwhoclassification.html).

b Low stage: 0‐II; high stage: III, IV (https://cancerstaging.org/Pages/default.aspx).

There were no statistical differences between diabetics and nondiabetics in the pathological variables distribution (Table S1).

### 3.2 Protein extraction from FFPE samples and differential quantitation by iTRAQ‐LC‐MS/MS

Proteins were extracted from FFPE. As expected, significantly higher protein concentrations were obtained from tumor tissue than from normal mucosa, both for diabetics and nondiabetics. No significant differences in protein extraction were observed when comparing diabetics and nondiabetics, either in tumor tissue or in mucosa (Fig. S2).

Eight‐plex quantitative proteomic analyses included two biological replicates per condition: diabetic and nondiabetic tumor tissue (TD and T, respectively), and their adjacent diabetic and nondiabetic normal mucosa (ND and N, respectively). Each biological replicate was labeled as shown in Fig. S1. No bias was introduced among labeled samples in terms of tumor site (right or left) or treatment in case of T2DM. To increase proteome coverage, sensitivity, and high‐throughput capacity, we performed a shotgun proteomics analysis and the peptide mixture of labeled samples was fractionated in 15 fractions prior to LC‐MS/MS. Table S2 shows the number of acquired MS/MS spectra, the total number of identified PSMs for a protein, and the number of peptides and proteins identified per fraction.

### 3.3 Identification of protein signatures associated with diabetes in tumor and normal colon

**Figure 1:**
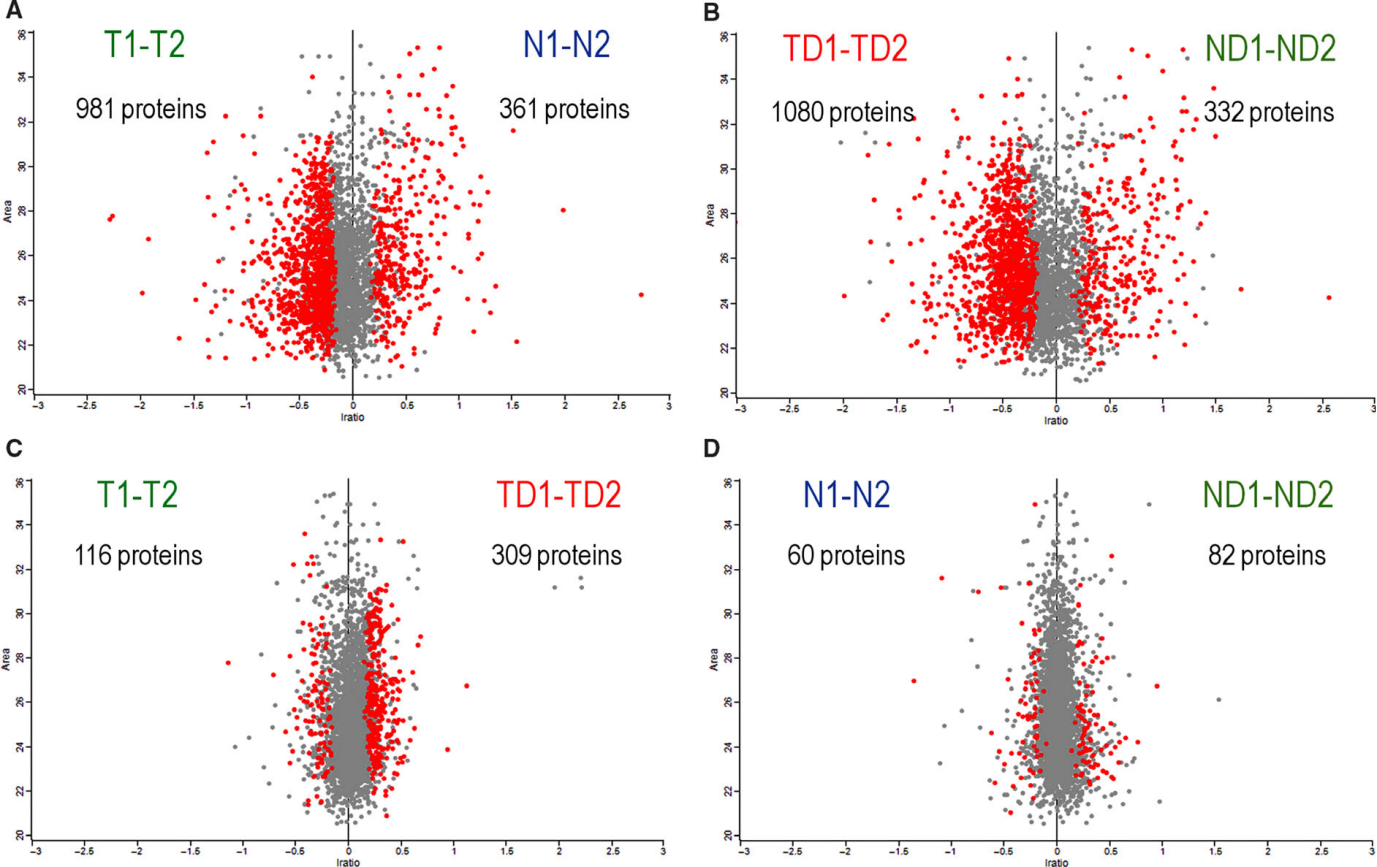
Statistical analyses of identified proteins resulted in subsets of proteins differentially expressed between compared conditions (T, N, TD, and ND) represented as red dots. (A) Nine hundred eighty‐one proteins upregulated in T versus N. (B) One thousand eighty proteins upregulated in TD versus ND. (C) Three hundred nine proteins upregulated in TD versus T. (D) Eighty‐two proteins upregulated in ND versus N.

From a total of 3076 identified proteins (Table S3), 1342 proteins were significantly altered in nondiabetic tumor tissue (T) compared with normal adjacent mucosa (N): 981 upregulated (790 with a fold change > 1.2) and 361 downregulated (314 with a fold change > 1.2; Fig. 1A). In diabetics, 1412 showed altered levels: 1080 upregulated (991 with a fold change > 1.2) and 332 downregulated (302 fold change > 1.2) in tumor (TD) compared with mucosa (ND; Fig. 1B). When evaluating a potential effect of T2DM on the CC tissue proteome, we found 425 proteins differentially expressed in diabetics compared with nondiabetics at tumor area (TD versus T; Fig. 1C), 309 upregulated (175 fold change > 1.2, Table S4), and 116 downregulated (76 fold change > 1.2, Table S5). Interestingly, T2DM also influenced the proteome in the adjacent mucosa: 143 proteins were differentially expressed, 82 upregulated (51 with fold change > 1.2, Table S4), and 60 downregulated (26 with fold change > 1.2, Table S5) in ND versus N (Fig. 1D). From these comparisons, we selected proteins up‐ and downregulated with FDR < 0.05 and fold change > 1.2 (log(2) = 0.26) for further analysis.

### 3.4 Proteins related to mitochondrial activity are key targets of T2DM in both tumor and normal adjacent mucosa

**Figure 2:**
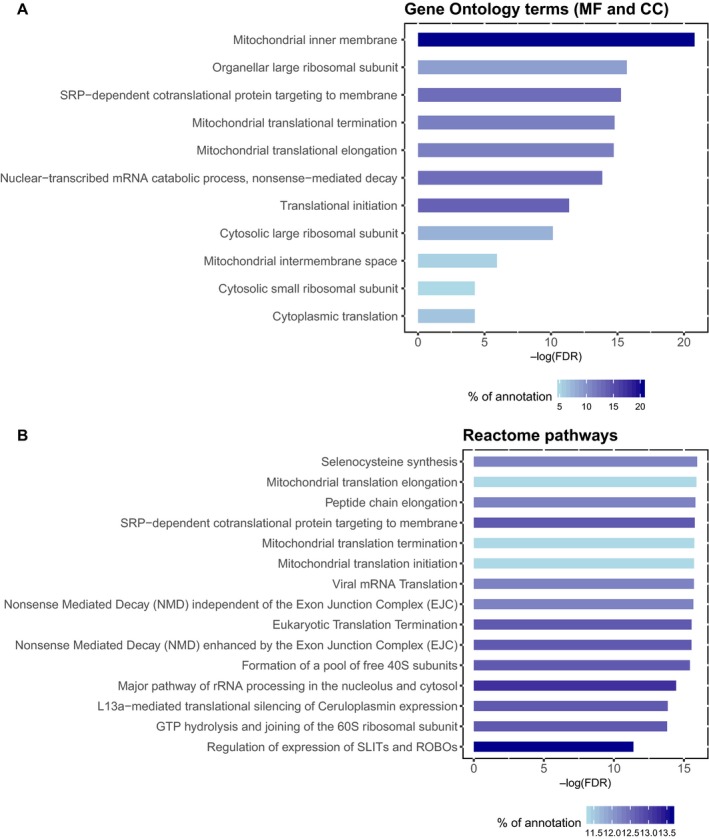
Functional enrichment of the proteins upregulated in diabetics in both the tumor and the normal colonic mucosa. (A) The most specific (nonredundant) Gene Ontology terms from biological processes (BP) and cellular component (CC) with FDR < 0.05. (B) The most specific (nonredundant) Reactome pathways with FDR < 0.05. In both plots, the terms are sorted according to their statistical significance (FDR) in log scale. The intensity of the gray scale colors in the bars shows the percentage of annotation with every term in the set of proteins selected as upregulated in diabetics.

A total of 220 proteins were upregulated in diabetics in either the tumor or its adjacent mucosa. An enrichment analysis of this signature provides several over‐represented Gene Ontology terms and Reactome pathways (Table S6). The main nonredundant terms are shown in Fig. 2 with three keywords in common: mitochondria, ribosome, and translation. Interestingly, there are only six proteins upregulated both in tumor and mucosa in diabetics, five of them are mitochondrial proteins (Table 2). In contrast to the strong signal coming from the upregulation toward mitochondrial dysfunction, the downregulated proteins showed a more heterogeneous scenario in terms of affected functions in both the common proteins downregulated in diabetic tumors and mucosas (Table S7) and the over‐represented functions in the whole set (Fig. S3 and Table S8).

**Table 2:** Proteins upregulated in diabetics versus nondiabetics, both in tumor and in adjacent mucosa.

Entry	Protein id	Protein Name	Tumor Diabetic to nondiabetic ratio (log2)	Mucosa Diabetic to nondiabetic ratio (log2)
O14602	EIF1AY	Eukaryotic translation initiation factor 1A, Y‐chromosomal	1.126	0.954
Q96EY8	MMAB	Cob(I)yrinic acid a,c‐diamide adenosyltransferase, mitochondrial	0.631	0.442
Q9Y5J9	TIMM8B	Mitochondrial import inner membrane translocase subunit Tim8B	0.471	0.769
Q9H0U6	MRPL18	39S ribosomal protein L18, mitochondrial	0.420	0.339
Q96EL3	MRPL53	39S ribosomal protein L53, mitochondrial	0.404	0.258
O60220	TIMM8A	Mitochondrial import inner membrane translocase subunit Tim8 A	0.303	0.268

**Figure 3:**
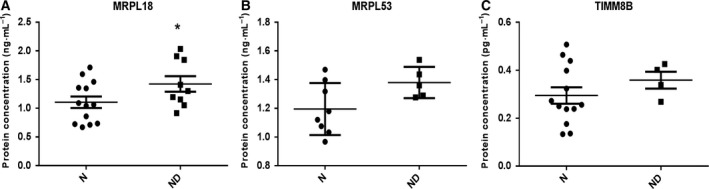
ELISA analysis for MRPL18 (A), MRPL53 (B), and TIMM8B (C) in individual extracts from T and N. *P‐value < 0.05.

Since both the mucosa and the tumor share this molecular signature, we focused on mucosa samples for confirmation analysis with the premise that these shared features between mucosa and tumor could represent the earliest stage of carcinogenesis. Analysis of individual extracts (not pools) by ELISA confirmed the increased expression in ND compared with N for the mitochondrial proteins MRPL18 (Fig. 3A), MRPL53 (Fig. 3B), and TIMM8B (Fig. 3C).

**Figure 4:**
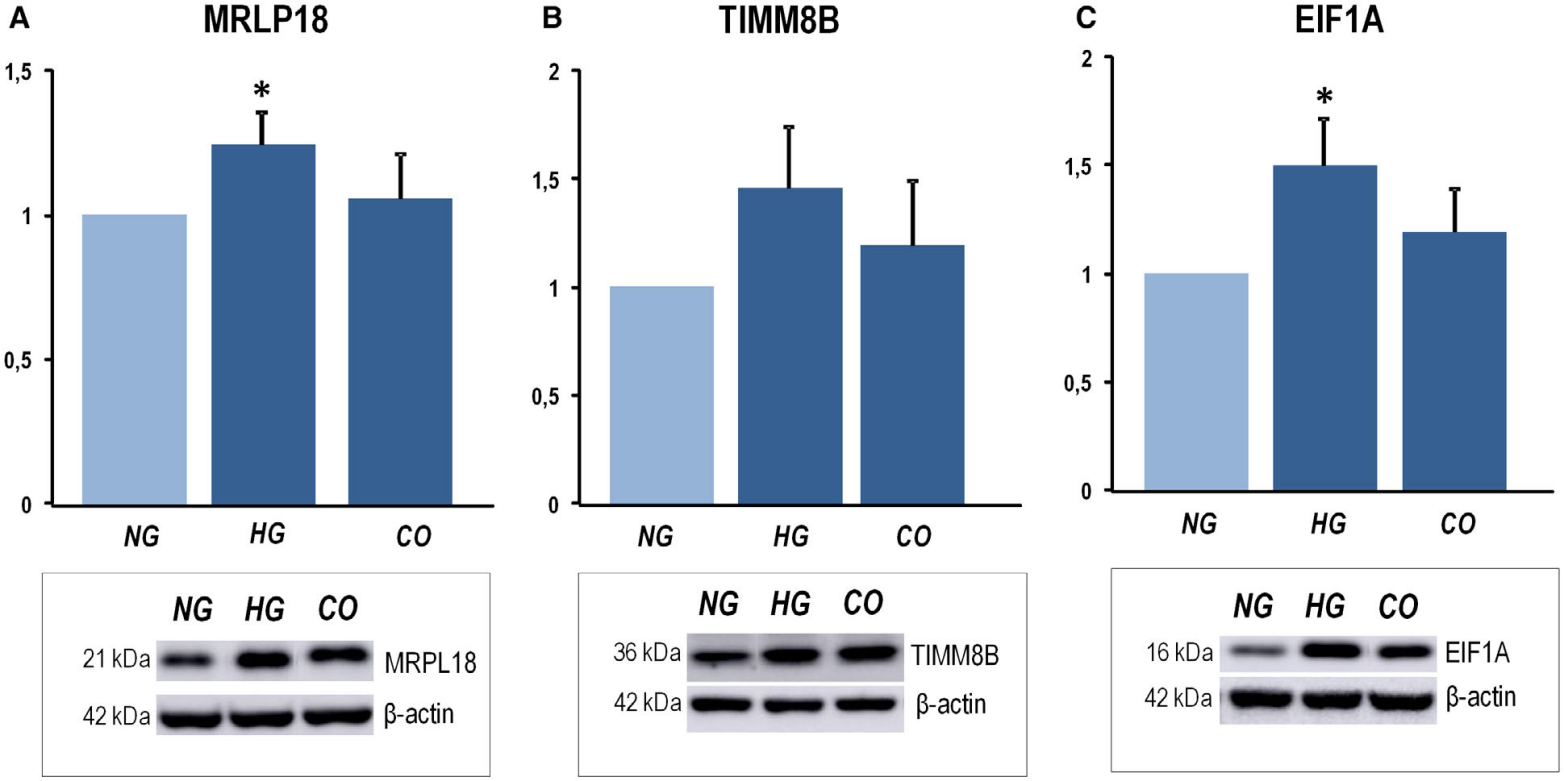
Western blot analysis of MRPL18 (A), TIMM8B (B), and EIF1A (C) from *in vitro* experiments using a normal colon mucosa epithelial cell line exposed to high (HG) or NG condition. Relative densitometric quantification and representative immunoblots are shown. CO: (osmotic) control. Error bars indicate SEM. Mann–Whitney statistical *U*‐test used with three biologically independent replicates included (*P‐value < 0.05).

Additionally, for further validation, cultured epithelial cell line derived from normal colon mucosa was exposed to high or normal glucose (NG) condition. In agreement with MS data, western blot analysis of cell extracts revealed higher levels of MRPL18 (Fig. 4A), TIMM8B (Fig. 4B), and EIF1A (Fig. 4C) in high glucose than in NG, and no changes in the osmotic control condition. This shows that high microenvironmental glucose levels, a cardinal feature of diabetes, could itself elicit the differential expression of at least some proteins identified as differentially regulated by diabetes *in vivo*.

## 4 Discussion

The FC concept, defined as the capacity of initiation of carcinogenesis processes in a normal tissue exposed to some factors such as inflammatory processes, has already been demonstrated using proteomics (Galandiuk *et al.*, 2012; Leedham *et al.*, 2009). To the best of our knowledge, here we show for the first time specific molecular changes triggered by T2DM in CC patients. These changes represent evidence of FC due to diabetes represented by abnormal protein levels in tumor and normal adjacent mucosa. In this regard, we identified six proteins upregulated in the tumor region and its adjacent normal mucosa triggered by T2DM.

This protein signature reveals a diabetes‐associated abnormal mitochondrial protein pattern, which is in agreement with previously described impaired mitochondrial responses to metabolic regulation in insulin resistance (Sivitz and Yorek, 2010). Supporting this concept, proteins associated with mitochondrial energy metabolism had been identified when compared diabetic and nondiabetic transgenic mice (Lu *et al.*, 2008). Indeed, mitochondrial abnormalities may result in increased reactive oxygen species production, causing subsequent organ damage (Szendroedi *et al.*, 2011). In this regard, higher mitochondrial mass, increased mitobiogenesis, and translation were positively correlated with proliferating tissue compared with solid (stable) tumors, and in metastatic tumors, they correlated with recurrence and poor clinical outcome (Hüttemann *et al.*, 2007). Gene expression studies have identified differential expression of mitochondrial ribosomes (mitoribosomes) genes in association with cancer development, and mitochondria have been proposed as key targets in cancer therapeutics (Järås and Ebert, 2011; Kim *et al.*, 2017; Koc *et al.*, 2015; Lamb *et al.*, 2015; Wallace, 2012). In particular, more than 95 gene transcripts associated with mitochondrial biogenesis and/or translation were significantly upregulated in human epithelial breast cancer cells. Of them, > 40% were mitochondrial ribosomal proteins (MRPs) including MRPL18 and TIMM8B (Sotgia *et al.*, 2012) that we here report to be upregulated in diabetics both in the tumor and the mucosa. MRPs are proteins functionally associated with mitochondrial translation of OXPHOS complex components, and with these evidences, we may hypothesize that a diabetic environment may also amplify oxidative mitochondrial metabolism and favor mitochondrial biogenesis similarly to what was observed in epithelial cancer cells. In a different study, high gene expression of a subset of MRPs including MRPL18 correlated with poor breast cancer survival (Huang *et al.*, 2015), and predicted tumor recurrence and tamoxifen resistance (Sotgia *et al.*, 2017). A new role for MRPL18 has been reported in the cytosolic stress response, in agreement with the diabetic microenvironment (Zhang *et al.*, 2015). EIF1A is a eukaryotic translation initiation factor. As a PAS kinase substrate, it regulates the cellular metabolism and glucose homeostasis (DeMille and Grose, 2013). An altered translation initiation is increasingly related to the risk of cancer, with a potential impact of EIF1A on NF‐kB signaling in an inflammatory microenvironment (Spilka). Finally, TIMM8A is a mitochondrial import inner membrane translocase subunit and its increase was related to increased malignancy in what refers to proliferation, metastasis, or cellular migration (Arnoult *et al.*, 2005; Gagné *et al.*, 2007; Peiris‐Pagès *et al.*, 2018; Rosdah *et al.*, 2016).

We present herein a proteomic experiment with four types of paired samples from CC patients divided into diabetics and nondiabetics. Although we have focused on the description of the diabetes effect in normal colonic mucosa and the signal that shares with the tumor, our rich framework can be exploited for discovery in several fronts. For instance, we found Fumarate hydratase, again a mitochondrial protein, downregulated in tumors versus normal mucosa in nondiabetics supporting the Warburg effect and Peto's paradox (Peto, 2016; Tidwell *et al.*, 2017; Warburg, 1956).

The present study has some limitations. The number of studied samples was not high. However, the patients were carefully selected from a wider cohort for homogeneity and comparability. Additionally, although the identified differentially expressed mitochondrial are known to be involved in tumor development, the definitive assignment of a cause‐and‐effect relationship to FC the diabetes context and to the increased risk of CC in diabetes requires carefully designed interventional experimental approaches.

## 5 Conclusions

We describe for the first time an altered proteome suggestive of mitochondrial deregulation in diabetic CC patients, thus supporting the epidemiological evidence of a higher risk of cancer development in diabetics. Since some of the identified proteins are known to be involved in cancer, the findings are consistent with the FC concept in a diabetic environment. The mitochondrial protein signature identifies a potential therapeutic target that should be further explored regarding its potential contribution to the higher risk of CC in diabetics.

## Conflict of interest

The authors declare no conflict of interest.

## Author contributions

LDP, PM, and GAL wrote the manuscript and researched data. LDP, PM, GAL, FR, JE, and JGF conceived and designed the experiments. ASH, SSC, NG, MR, MC, and IP researched data. SM, ABS, OA, CGG, CA, AO, FR, JE, and JGF contributed to discussion and reviewed/edited the manuscript. All authors approved the final version of the manuscript.

## Supporting information


**Fig. S1**. Isobaric labeling experiment (iTRAQ).
**Fig. S2**. Concentration of protein extracted from FFPE samples of tumor and mucosa from diabetics and nondiabetics.
**Fig. S3**. Functional enrichment of the proteins downregulated in diabetics in both the tumor and the normal colonic mucosa.
**Table S1**. Mutational, immunohistochemical and proliferation characterization of CC samples.
**Table S2**. Number of acquired MS/MS spectra, the total number of identified PSMs for a protein, and the number of peptides and proteins identified per fraction.

**Table S3**. Repository of identified proteins.
**Table S4**. Lists of upregulated proteins in diabetic tumors and mucosas (compared to nondiabetic tumors and mucosas respectively).
**Table S5**. Lists of downregulated proteins in diabetic tumors and mucosas (compared to nondiabetic tumors and mucosas respectively).

**Table S6**. Over‐represented Gene Ontology terms and Reactome pathways in upregulated protein in diabetics tumors and mucosas.
**Table S7**. Downregulated proteins in diabetics, common to tumor and normal adjacent mucosa.

**Table S8**. Over‐represented Gene Ontology terms and Reactome pathways in downregulated protein in diabetics tumors and mucosas.

## References

[mol212531-bib-0001] Arnoult D , Rismanchi N , Grodet A , Roberts RG , Seeburg DP , Estaquier J , Sheng M and Blackstone C (2005) Bax/Bak‐dependent release of DDP/TIMM8a promotes Drp1‐mediated mitochondrial fission and mitoptosis during programmed cell death. Curr Biol 15, 2112–2118. 16332536 10.1016/j.cub.2005.10.041

[mol212531-bib-0002] Cardoso J , Boer J , Morreau H and Fodde R (2007) Expression and genomic profiling of colorectal cancer. Biochim Biophys Acta 1775, 103–137. 17010523 10.1016/j.bbcan.2006.08.004

[mol212531-bib-0003] Curtius K , Wright NA and Graham TA (2018) An evolutionary perspective on field cancerization. Nat Rev Cancer 18, 19–32. 29217838 10.1038/nrc.2017.102

[mol212531-bib-0004] de Kort S , Simons CC , van den Brandt PA , Goldbohm RA , Arts IC , de Bruine AP , Janssen‐Heijnen ML , Sanduleanu S , Masclee AA and Weijenberg MP (2016) Diabetes mellitus type 2 and subsite‐specific colorectal cancer risk in men and women: results from the Netherlands Cohort Study on diet and cancer. Eur J Gastroenterol Hepatol 28, 896–903. 27097356 10.1097/MEG.0000000000000626

[mol212531-bib-0005] Del Puerto‐Nevado L , Minguez P , Corton M , Solanes‐Casado S , Prieto I , Mas S , Sanz AB , Gonzalez‐Alonso P , Villaverde C , Portal‐Nuñez S *et al* (2019) Molecular evidence of field cancerization initiated by diabetes in colon cancer patients. Mol Oncol 13, 857–887. 30628165 10.1002/1878-0261.12438 PMC6441931

[mol212531-bib-0006] DeMille D and Grose JH (2013) PAS kinase: a nutrient sensing regulator of glucose homeostasis. IUBMB Life 65, 921–929. 24265199 10.1002/iub.1219 PMC4081539

[mol212531-bib-0007] Gagné JP , Ethier C , Gagné P , Mercier G , Bonicalzi ME , Mes‐Masson AM , Droit A , Winstall E , Isabelle M and Poirier GG (2007) Comparative proteome analysis of human epithelial ovarian cancer. Proteome Sci 5, 16. 17892554 10.1186/1477-5956-5-16 PMC2072939

[mol212531-bib-0008] Galandiuk S , Rodriguez‐Justo M , Jeffery R , Nicholson AM , Cheng Y , Oukrif D , Elia G , Leedham SJ , McDonald SA , Wright NA *et al* (2012) Field cancerization in the intestinal epithelium of patients with Crohn’s ileocolitis. Gastroenterology 142, 855–864.e8. 22178590 10.1053/j.gastro.2011.12.004 PMC4446968

[mol212531-bib-0009] Gámez‐Pozo A , Sánchez‐Navarro I , Ferrer NI , Martínez FG , Ashman K and Vara JÁF (2012) High‐throughput phosphoproteomics from formalin‐fixed, paraffin‐embedded tissues. Curr Protoc Chem Biol 4, 161–175.

[mol212531-bib-0010] Guo H , Zeng W , Feng L , Yu X , Li P , Zhang K , Zhou Z and Cheng S (2017) Integrated transcriptomic analysis of distance‐related field cancerization in rectal cancer patients. Oncotarget 8, 61107–61117. 28977850 10.18632/oncotarget.17864 PMC5617410

[mol212531-bib-0011] Harding JL , Shaw JE , Peeters A , Cartensen B and Magliano DJ (2015) Cancer risk among people with type 1 and type 2 diabetes: disentangling true associations, detection bias, and reverse causation. Diabetes Care 38, 264–270. 25488912 10.2337/dc14-1996

[mol212531-bib-0012] Huang Z , Duan H and Li H (2015) Identification of gene expression pattern related to breast cancer survival using integrated TCGA datasets and genomic tools. Biomed Res Int 2015, 1–10. 10.1155/2015/878546 PMC4630377 26576432

[mol212531-bib-0013] Hüttemann M , Lee I , Samavati L , Yu H and Doan JW (2007) Regulation of mitochondrial oxidative phosphorylation through cell signaling. Biochim Biophys Acta 1773, 1701–1720. 18240421 10.1016/j.bbamcr.2007.10.001

[mol212531-bib-0014] Järås M and Ebert BL (2011) Power cut: inhibiting mitochondrial translation to target leukemia. Cancer Cell 20, 555–556. 22094249 10.1016/j.ccr.2011.10.028

[mol212531-bib-0015] Jimenez CR , Knol JC , Meijer GA and Fijneman RJA (2010) Proteomics of colorectal cancer: overview of discovery studies and identification of commonly identified cancer‐associated proteins and candidate CRC serum markers. J Proteomics 73, 1873–1895. 20601272 10.1016/j.jprot.2010.06.004

[mol212531-bib-0016] Johnson JA , Carstensen B , Witte D , Bowker SL , Lipscombe L , Renehan AG ; Diabetes and Cancer Research Consortium (2012) Diabetes and cancer (1): evaluating the temporal relationship between type 2 diabetes and cancer incidence. Diabetologia 55, 1607–1618. 22476947 10.1007/s00125-012-2525-1

[mol212531-bib-0017] Kim H‐J , Maiti P and Barrientos A (2017) Mitochondrial ribosomes in cancer. Semin Cancer Biol 47, 67–81. 28445780 10.1016/j.semcancer.2017.04.004 PMC5662495

[mol212531-bib-0018] Koc EC , Haciosmanoglu E , Claudio PP , Wolf A , Califano L , Friscia M , Cortese A and Koc H (2015) Impaired mitochondrial protein synthesis in head and neck squamous cell carcinoma. Mitochondrion 24, 113–121. 26238294 10.1016/j.mito.2015.07.123

[mol212531-bib-0019] Lamb R , Ozsvari B , Lisanti CL , Tanowitz HB , Howell A , Martinez‐Outschoorn UE , Sotgia F and Lisanti MP (2015) Antibiotics that target mitochondria effectively eradicate cancer stem cells, across multiple tumor types: treating cancer like an infectious disease. Oncotarget 6, 4569–4584. 25625193 10.18632/oncotarget.3174 PMC4467100

[mol212531-bib-0020] Leedham SJ , Graham TA , Oukrif D , McDonald SA , Rodriguez‐Justo M , Harrison RF , Shepherd NA , Novelli MR , Jankowski JA and Wright NA (2009) Clonality, founder mutations, and field cancerization in human ulcerative colitis‐associated neoplasia. Gastroenterology 136, 542–550.e6. 19103203 10.1053/j.gastro.2008.10.086

[mol212531-bib-0021] Lu H , Yang Y , Allister EM , Wijesekara N and Wheeler MB (2008) The identification of potential factors associated with the development of type 2 diabetes: a quantitative proteomics approach. Mol Cell Proteomics 7, 1434–1451. 18448419 10.1074/mcp.M700478-MCP200 PMC2500228

[mol212531-bib-0022] Mi H , Muruganujan A and Thomas PD (2013) PANTHER in 2013: modeling the evolution of gene function, and other gene attributes, in the context of phylogenetic trees. Nucleic Acids Res 41, D377–D386. 23193289 10.1093/nar/gks1118 PMC3531194

[mol212531-bib-0023] Nambiar PR , Gupta RR and Misra V (2010) An “Omics” based survey of human colon cancer. Mutat Res 693, 3–18. 20691711 10.1016/j.mrfmmm.2010.07.008

[mol212531-bib-0024] Peiris‐Pagès M , Bonuccelli G , Sotgia F and Lisanti MP (2018) Mitochondrial fission as a driver of stemness in tumor cells: mDIVI1 inhibits mitochondrial function, cell migration and cancer stem cell (CSC) signalling. Oncotarget 9, 13254–13275. 29568355 10.18632/oncotarget.24285 PMC5862576

[mol212531-bib-0025] Peto R (2016) Epidemiology, multistage models, and short‐term mutagenicity tests. Int J Epidemiol 45, 621–637. 27582437 10.1093/ije/dyv199

[mol212531-bib-0026] Prieto I , Del Puerto-Nevado L , Gonzalez N , Portal-Nuñez S , Zazo S , Corton M , Minguez P , Gomez-Guerrero C , Arce JM , Sanz AB *et al* (2017) Colon cancer modulation by a diabetic environment: a single institutional experience. PLoS One 12, e0172300. 28253286 10.1371/journal.pone.0172300 PMC5333811

[mol212531-bib-0027] Renehan AG , Yeh H‐C , Johnson JA , Wild SH , Gale EAM and Møller H ; Diabetes and Cancer Research Consortium (2012) Diabetes and cancer (2): evaluating the impact of diabetes on mortality in patients with cancer. Diabetologia 55, 1619–1632. 22476948 10.1007/s00125-012-2526-0

[mol212531-bib-0028] Rosdah AA , Holien KJ , Delbridge LMD , Dusting GJ and Lim SY (2016) Mitochondrial fission ‐ a drug target for cytoprotection or cytodestruction? Pharmacol Res Perspect 4, e00235. 27433345 10.1002/prp2.235 PMC4876145

[mol212531-bib-0029] Rubin H (2013) Promotion and selection by serum growth factors drive field cancerization, which is anticipated *in vivo* by type 2 diabetes and obesity. Proc Natl Acad Sci USA 110, 13927–13931. 23908399 10.1073/pnas.1312831110 PMC3752243

[mol212531-bib-0030] Sanz‐Pamplona R , Berenguer A , Cordero D , Molleví DG , Crous‐Bou M , Sole X , Paré‐Brunet L , Guino E , Salazar R , Santos C * et al* (2014) Aberrant gene expression in mucosa adjacent to tumor reveals a molecular crosstalk in colon cancer. Mol Cancer 13, 46. 24597571 10.1186/1476-4598-13-46 PMC4023701

[mol212531-bib-0031] Sivitz WI and Yorek MA (2010) Mitochondrial dysfunction in diabetes: from molecular mechanisms to functional significance and therapeutic opportunities. Antioxid Redox Signal 12, 537–577. 19650713 10.1089/ars.2009.2531 PMC2824521

[mol212531-bib-0032] Slaughter DP , Southwick HW and Smejkal W (1953) Field cancerization in oral stratified squamous epithelium; clinical implications of multicentric origin. Cancer 6, 963–968. 13094644 10.1002/1097-0142(195309)6:5<963::aid-cncr2820060515>3.0.co;2-q

[mol212531-bib-0033] Sotgia F , Fiorillo M and Lisanti MP (2017) Mitochondrial markers predict recurrence, metastasis and tamoxifen‐resistance in breast cancer patients: Early detection of treatment failure with companion diagnostics. Oncotarget 8, 68730–68745. 28978152 10.18632/oncotarget.19612 PMC5620292

[mol212531-bib-0034] Sotgia F , Whitaker‐Menezes D , Martinez‐Outschoorn UE , Salem AF , Tsirigos A , Lamb R , Sneddon S , Hulit J , Howell A and Lisanti MP (2012) Mitochondria “fuel” breast cancer metabolism: fifteen markers of mitochondrial biogenesis label epithelial cancer cells, but are excluded from adjacent stromal cells. Cell Cycle 11, 4390–4401. 23172368 10.4161/cc.22777 PMC3552922

[mol212531-bib-0035] Suh S and Kim K‐W (2011) Diabetes and cancer: is diabetes causally related to cancer? Diabetes Metab J 35, 193–198. 21785737 10.4093/dmj.2011.35.3.193 PMC3138100

[mol212531-bib-0036] Szendroedi J , Phielix E and Roden M (2011) The role of mitochondria in insulin resistance and type 2 diabetes mellitus. Nat Rev Endocrinol 8, 92–103. 21912398 10.1038/nrendo.2011.138

[mol212531-bib-0037] Tidwell TR , Søreide K and Hagland HR (2017) Aging, metabolism, and cancer development: from Peto’s paradox to the warburg effect. Aging Dis 8, 662–676. 28966808 10.14336/AD.2017.0713 PMC5614328

[mol212531-bib-0038] Uzozie A , Nanni P , Staiano T , Grossmann J , Barkow‐Oesterreicher S , Shay JW , Tiwari A , Buffoli F , Laczko E and Marra G (2014) Sorbitol dehydrogenase overexpression and other aspects of dysregulated protein expression in human precancerous colorectal neoplasms: a quantitative proteomics study. Mol Cell Proteomics 13, 1198–1218. 24567419 10.1074/mcp.M113.035105 PMC4014279

[mol212531-bib-0039] Wallace DC (2012) Mitochondria and cancer. Nat Rev Cancer 12, 685–698. 23001348 10.1038/nrc3365 PMC4371788

[mol212531-bib-0040] Wang Y , Yang F , Gritsenko MA , Wang Y , Clauss T , Liu T , Shen Y , Monroe ME , Lopez‐Ferrer D , Reno T * et al* (2011) Reversed‐phase chromatography with multiple fraction concatenation strategy for proteome profiling of human MCF10A cells. Proteomics 11, 2019–2026. 21500348 10.1002/pmic.201000722 PMC3120047

[mol212531-bib-0041] Warburg O (1956) On the origin of cancer cells. Science 123, 309–314. 13298683 10.1126/science.123.3191.309

[mol212531-bib-0042] Wiśniewski JR , Duś‐Szachniewicz K , Ostasiewicz P , Ziółkowski P , Rakus D and Mann M (2015) Absolute proteome analysis of colorectal mucosa, adenoma, and cancer reveals drastic changes in fatty acid metabolism and plasma membrane transporters. J Proteome Res 14, 4005–4018. 26245529 10.1021/acs.jproteome.5b00523

[mol212531-bib-0043] Wiśniewski JR , Ostasiewicz P , Duś K , Zielińska DF , Gnad F and Mann M (2012) Extensive quantitative remodeling of the proteome between normal colon tissue and adenocarcinoma. Mol Syst Biol 8, 611. 22968445 10.1038/msb.2012.44 PMC3472694

[mol212531-bib-0044] Wiśniewski JR , Zougman A , Nagaraj N and Mann M (2009) Universal sample preparation method for proteome analysis. Nat Methods 6, 359–362. 19377485 10.1038/nmeth.1322

[mol212531-bib-0045] Zhang X , Gao X , Coots RA , Conn CS , Liu B and Qian S‐B (2015) Translational control of the cytosolic stress response by mitochondrial ribosomal protein L18. Nat Struct Mol Biol 22, 404–410. 25866880 10.1038/nsmb.3010 PMC4424103

