# Supplementary material for: Diabetes‐mediated promotion of colon mucosa carcinogenesis is associated with mitochondrial dysfunction
Source: Mol Oncol. 2019 Jul 27;13(9):1887–97. doi: 10.1002/1878-0261.12531 (PMC6717745; doi:10.1002/1878-0261.12531)
Supplement: Supplementary file 1 — Fig. S1. Isobaric labeling experiment (iTRAQ). Fig. S2. Concentration of protein extracted from FFPE samples of tumor and mucosa from diabetics and nondiabetics. Fig. S3. Functional enrichment of the proteins downregulated in diabetics in both the tumor and the normal colonic mucosa. Table S1. Mutational, immunohistochemical and proliferation characterization of CC samples. Table S2. Number of acquired MS/MS spectra, the total number of identified PSMs for a protein, and the number of peptides and proteins identified per fraction. [file MOL2-13-1887-s001.pdf]

SUPPLEMENTARY INFORMATION

Diabetes promotion of colon mucosa carcinogenesis is associated to mitochondrial dysfunction

Laura Del Puerto-Nevado [1], Aranzazu Santiago-Hernandez [2], Sonia Solanes-Casado [1], Nieves Gonzalez [3], Marta Ricote [3], Marta Corton [4,5], Isabel Prieto [6], Sebastian Mas [3], Ana Belen Sanz [7,8], Oscar Aguilera [1], Carmen Gomez-Guerrero [3], Carmen Ayuso [4,5], Alberto Ortiz [7,8], Federico Rojo [9], Jesus Egido [3], Jesus Garcia-Foncillas [1], Pablo Minguez [4*], Gloria Alvarez-Llamas [2,8#], on behalf of the DiabetesCancerConnect Consortium.

1 Translational Oncology Division, Oncohealth Institute, IIS-Fundacion Jimenez Diaz-UAM, Madrid, Spain.
2 Immunology Department, IIS-Fundacion Jimenez Diaz-UAM, Madrid, Spain.
3 Renal, Vascular and Diabetes Research Laboratory, IIS-Fundacion Jimenez Diaz-UAM, Spanish Biomedical Research Network in Diabetes and Associated Metabolic Disorders (CIBERDEM), Madrid, Spain.
4 Genetics Department, IIS-Fundacion Jimenez Diaz-UAM, Madrid, Spain.
5 Center for Biomedical Network Research on Rare Diseases (CIBERER), ISCIII, Madrid, Spain.
6 Radiation Oncology, Oncohealth Institute, IIS-Fundacion Jimenez Diaz-UAM, Madrid, Spain.
7 Nephrology and Hypertension Department, IIS-Fundacion Jimenez Diaz-UAM, Madrid, Spain.
8 REDINREN, Madrid, Spain.
9 Pathology Department, IIS-Fundacion Jimenez Diaz-UAM, Madrid, Spain.

AND * Corresponding authors: galvarez@fjd.es, pablo.minguez@quironsalud.es

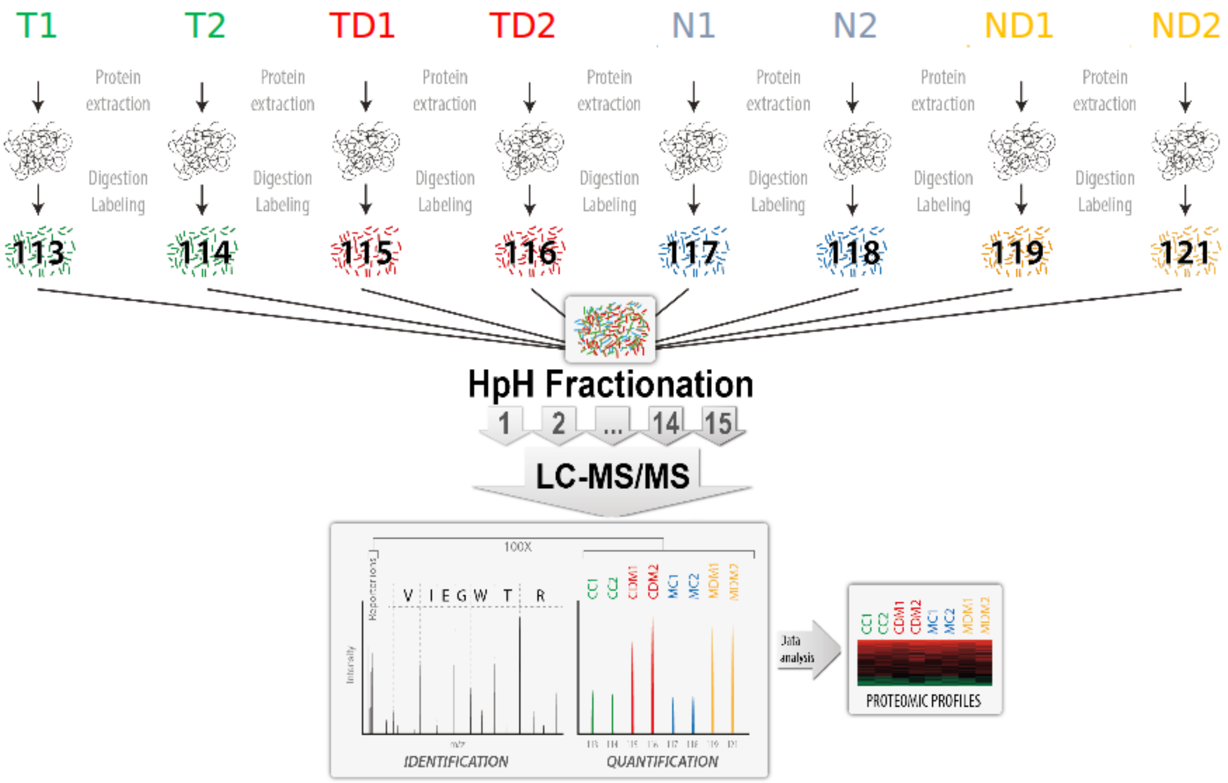

Supplementary Figure 1. Isobaric labeling experiment (iTRAQ). Two biological replicates were included per condition (T, TD, N and ND). Following labeling, the peptide mixture was fractionated and analyzed by LC-MS/MS. T: non-diabetic tumor, TD: diabetic tumor, N: no diabetic mucosa, ND: diabetic mucosa, LC: liquid chromatography, MS/MS: mass spectrometry in tandem. 113 to 121 are the reporter ions / isobaric tags.

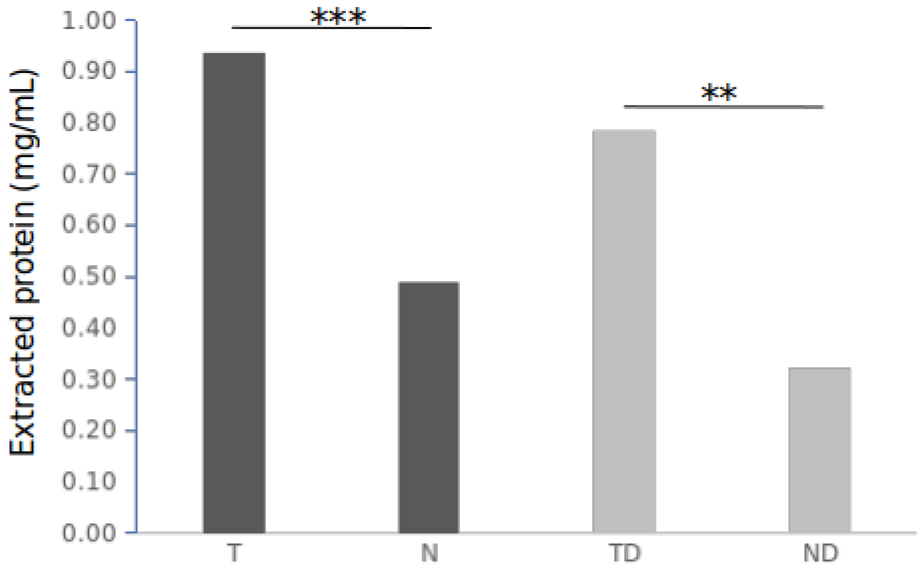

Supplementary Figure 2. Concentration of protein extracted from FFPE samples of tumor and mucosa from diabetics and non-diabetics. T: non-diabetic tumor, N: non-diabetic mucosa, TD: diabetic tumor, ND: diabetic mucosa, **p-value < 0.01, ***p-value <0.001.

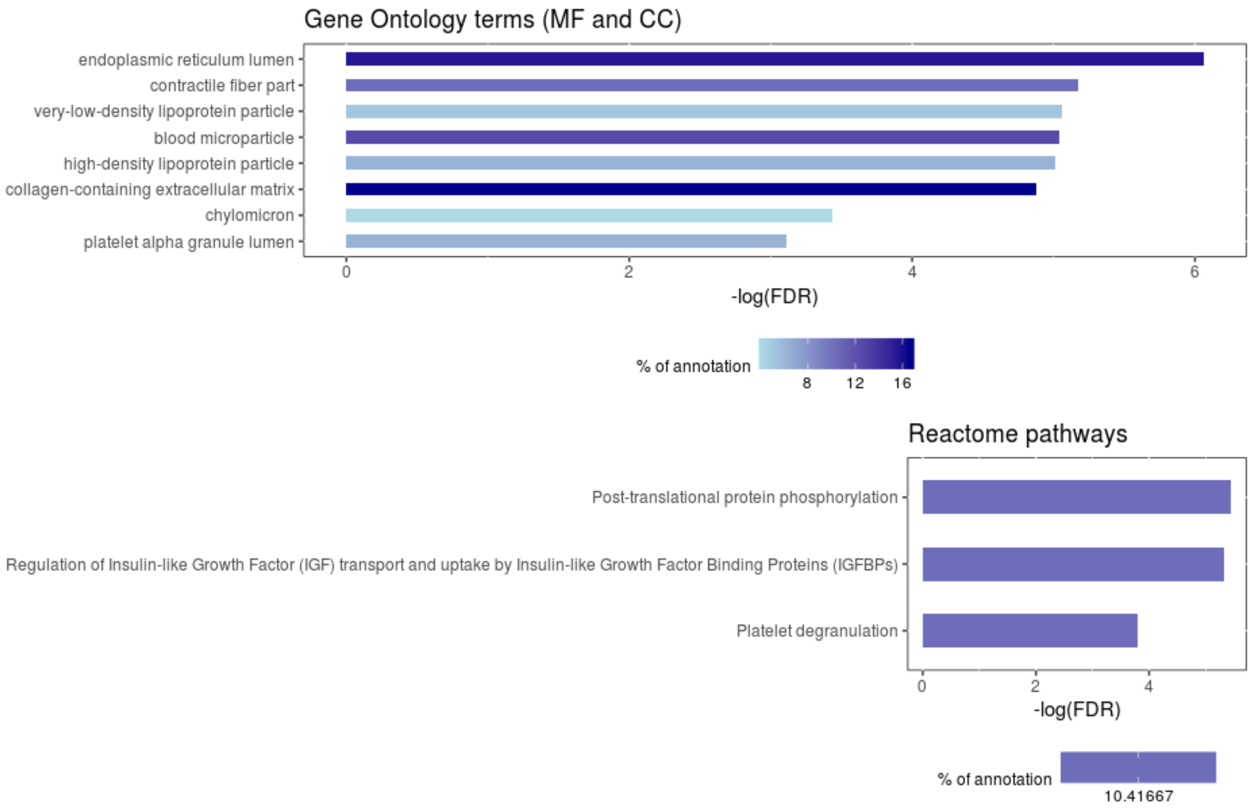

Supplementary Figure 3. Functional enrichment of the proteins down-regulated in diabetics in both the tumor and the normal colonic mucosa. A represents the most specific (non-redundant) Gene Ontology terms from biological processes (BP) and cellular component (CC) with FDR < 0.05. B represents the most specific (non-redundant) Reactome pathways with FDR < 0.05. In both plots, the terms are sorted according to their statistical significance (FDR) in log scale. The intensity of

the grey scale colors in the bars shows the percentage of annotation with every term in the set of proteins selected as up-regulated in diabetics.

Supplementary Table 1. Mutational, immunohistochemical and proliferation characterization of CC samples. P-value was calculated by Fisher's Exact test. IHC: immunohistochemistry.

**Gene sequencing**	**Mutant**	**Mutant**	**p-value**
KRAS	6	6	0.7
NRAS	1	0	1
BRAF	2	1	1
**IHC of MMR genes**	**Positive**	**Positive**	**p-value**
MLH1	11	12	0.05
MSH2	16	12	1
MSH6	16	12	1
PSM2	11	12	0,05
**Proliferation analysis**	**High**	**High**	**p-value**
Ki-67	10	9	1

Supplementary Table 2. Number of acquired MS/MS spectra, the total number of identified PSMs for a protein, and the number of peptides and proteins identified per fraction.

		MS/MS	PSM	Peptides	Proteins
20160621_EZ_E01_Gloria_FJD_8plex_F01	F1	21993	4167	2817	985
20160621_EZ_E01_Gloria_FJD_8plex_F02	F2	21563	3091	2069	842
20160621_EZ_E01_Gloria_FJD_8plex_F03	F3	21636	2935	1999	820
20160621_EZ_E01_Gloria_FJD_8plex_F04	F4	22279	2946	1934	793
20160621_EZ_E01_Gloria_FJD_8plex_F05	F5	21482	3103	2044	817
20160621_EZ_E01_Gloria_FJD_8plex_F06	F6	23130	2859	1906	702
20160621_EZ_E01_Gloria_FJD_8plex_F07 no Y	F7	24881	2676	1836	714
20160621_EZ_E01_Gloria_FJD_8plex_F08	F8	24477	2778	1972	721
20160621_EZ_E01_Gloria_FJD_8plex_F09	F9	24415	2617	1807	691
20160621_EZ_E01_Gloria_FJD_8plex_F10	F10	26760	2675	1851	703
20160621_EZ_E01_Gloria_FJD_8plex_F11	F11	26393	3207	2253	791
20160621_EZ_E01_Gloria_FJD_8plex_F12	F12	25169	2660	1855	646
20160621_EZ_E01_Gloria_FJD_8plex_F13	F13	26227	3000	2104	701
20160621_EZ_E01_Gloria_FJD_8plex_F14	F14	24995	2397	1673	552
20160621_EZ_E01_Gloria_FJD_8plex_F15	F15	24525	2864	1934	649
Multiconsensus		359925	44024	19718	3076

ADDITIONAL SUPPLEMENTARY TABLES IN SEPARATE EXCEL DOCUMENTS.

Supplementary Table 3. Repository of identified proteins

Supplementary Table 4. Lists of up-regulated proteins in diabetic tumors and mucosas (compared to non-diabetic tumors and mucosas respectively).

Supplementary Table 5. Lists of down-regulated proteins in diabetic tumors and mucosas (compared to non-diabetic tumors and mucosas respectively).

Supplementary Table 6. Over-represented Gene Ontology terms and Reactome pathways in up-regulated protein in diabetics tumors and mucosas.

Supplementary Table 7. Down-regulated proteins in diabetics, common to tumor and normal adjacent mucosa. Values in ratio (log2).

Entry	Protein id	Protein Name	Tumor Diabetic to non-diabetic	Mucosa Diabetic to non-diabetic
P02647	APOA1	Apolipoprotein A-I	-0.431	-0.331
P23297	S100A1	Protein S100-A1	-0.526	-0.549
P52848	NDST1	Bifunctional heparan sulfate N-deacetylase/N-sulfotransferase 1	-0.438	-1.356
Q63ZY3	KANK2	KN motif and ankyrin repeat domain-containing protein 2	-0.261	-0.289
Q8N5H7	SH2D3C	SH2 domain-containing protein 3C	-0.333	-0.320
Q9Y3D6	FIS1	Mitochondrial fission 1 protein	-0.370	-0.352

ADDITIONAL SUPPLEMENTARY TABLES IN SEPARATE EXCEL DOCUMENTS.

Supplementary Table 8. Over-represented Gene Ontology terms and Reactome pathways in down-regulated protein in diabetics tumors and mucosas.
